# Terahertz liquid networks: a mesoscopic passive architecture for circumventing physical deadlocks in brain–computer interfaces

**DOI:** 10.1039/d6na00618c

**Published:** 2026-10-05

**Authors:** Chenyan Zhou, Xinran Ding, Jiamin Zhao, Xuanhao Gu, Zhengyang Xu

**Affiliations:** a School of Medicine, Nantong University Nantong 226019 P. R. China; b Nantong Institute of Technology Nantong 226018 P. R. China 2231110115@stmail.ntu.edu.cn

## Abstract

Brain–computer interfaces (BCIs) for major neurological disorders face physical and physiological limitations. Mechanical mismatch in rigid microelectrode arrays precipitates chronic immune rejection and irreversible central nervous system damage, whereas bandwidth scaling in traditional wireless links inevitably breaches the 1 °C tissue thermal safety threshold. To circumvent this deadlock among bandwidth, heat dissipation, and biocompatibility, this review proposes the “terahertz liquid network” architecture. Utilizing injectable plasmonic nanoparticles as free-floating relay nodes, this architecture fundamentally abolishes the mechanical shear stresses characteristic of conventional implant interfaces. Exploiting broadband and dielectric properties of terahertz (THz) waves, the network achieves cross-scale bioelectronic integration across both physical and physiological boundaries. For communication and decoding, the system bypasses the strong THz absorption barrier of brain tissue *via* non-line-of-sight multipath scattering and THz-photoacoustic cross-modal transduction. Integrating these mechanisms with passive backscattering fulfills the terabits-per-second (Tbps) absolute bandwidth requirements dictated by full-cortical, high-fidelity motor decoding. For neuromodulation, specific THz photon energies are directed at voltage-gated ion channels to induce targeted non-thermal resonance, mediating precise, subcellular-level interventions devoid of macroscopic current overflow. Through an analysis of typical clinical scenarios—namely, audiovisual perception reconstruction and physical neural injury rehabilitation—this review demonstrates the feasibility of this architecture in resolving the conflict between high-throughput concurrency and stringent physiological constraints. Ultimately, the terahertz liquid network reconfigures the neural interface paradigm from a macroscopic active solid-state to a mesoscopic passive liquid-state. This transition grants the system superior biocompatibility and endows it with disease-modifying potential targeting underlying pathologies, establishing a trajectory for next-generation BCIs.

## Abstract 2

The terahertz liquid network utilizes free-floating plasmonic nodes to circumvent BCI physical deadlocks, achieving subcellular non-thermal resonance and Tbps-level absolute bandwidth without breaching the 1 °C thermal safety limit.

## Graphical abstract

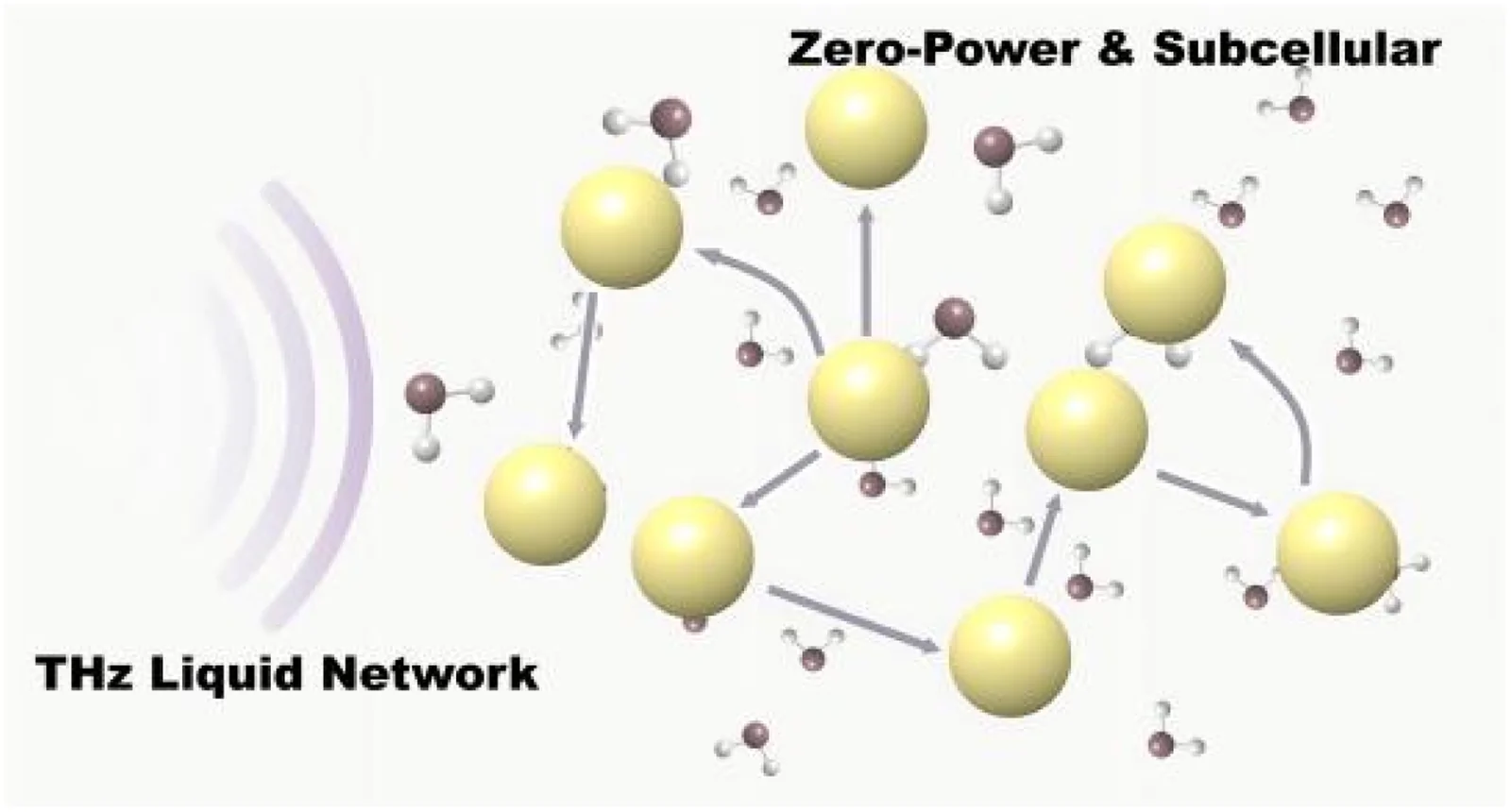

## 1. Introduction

Contemporary brain–computer interface (BCI) architectures remain restricted by a mutually antagonistic bottleneck comprising throughput, thermal safety, and biocompatibility. Dictated by the low-pass filtering effect of high-impedance cranial bone, non-invasive paradigms suffer from restricted spatiotemporal resolution, precluding sub-millimeter decoding precision.^1,2^ Although invasive microelectrode arrays elevate signal gain, the mechanical mismatch between rigid substrates and soft neural tissue inherently drives initial structural trauma.^3^ This persistent physical friction provokes acute neuroinflammation^4^ and triggers cascading reactive gliosis at the implant interface.^5^ The subsequent encapsulation by dense glial scars precipitates dynamic drifts in interfacial impedance, dictating the irreversible attenuation of chronic recording signals.^3^ Flexible polymer microelectrodes (*e.g.*, thread architectures) mitigate mechanical mismatch and scale channel capacities to the thousands ^6^ however, escalating concurrent data throughput dictates a proportional surge in radiofrequency (RF) power consumption, governed by the Shannon–Hartley theorem. Under the stringent volumetric constraints of micro-implants, restricted heat dissipation challenges extreme low-power baselines.^7^ Consequently, escalating data throughput readily breaches the strictly defined 1 °C thermal safety exposure threshold of brain tissue,^8^ thereby thermodynamically capping continuous high-frequency operations.^9^ Intravascular interfaces, while circumventing open cranial trauma, fail to resolve the inherent physical compromise between spatial sampling resolution and detection depth.^2^ Furthermore, traditional RF links, governed by the half-wavelength diffraction limit of classical electromagnetic waves, preclude miniaturization to mesoscopic or nanoscale dimensions. Expanding operational frequencies to minimize hardware footprint invariably encounters severe dipole resonance absorption by bulk tissue water,^10^ precipitating exponential signal dissipation across nanoscale biological communication channels.^11^ Forcibly compensating for such severe propagation attenuation *via* increased transmission power inevitably forces localized energy deposition beyond physiological thermal safety thresholds, provoking irreversible thermal damage.^8^ This triple physical deadlock—comprising RF bandwidth, antenna geometry, and tissue heat dissipation—indicates that traditional physical-contact BCI architectures are approaching their theoretical performance limits.^3,7^ Unique dielectric properties within the terahertz (THz) band—defined here as the electromagnetic spectrum ranging from 0.1 to 10 THz—offer a physical basis for reconstructing these underlying communication links.^5,12^ The broader biomedical utility of this spectrum has already been demonstrated by recent advances in high-sensitivity THz metamaterial absorbers and refractive index sensors.^13–16^ Building on these principles to circumvent the mechanical rejection and communication bottlenecks characteristic of conventional solid implants, this review introduces the “terahertz liquid network” conceptual framework. By deploying functionalized plasmonic nanoparticles as free-floating relay nodes, this paradigm resolves existing structural deficiencies and advances the scale of bioelectronic interaction from macroscopic charge recording to targeted kinematic interventions at the subcellular level.

Grounded in the complete biophysical transmission chain, this review systematically investigates the feasibility and translational trajectory of this novel paradigm. Section 2 elucidates the microscopic non-thermal resonance mechanisms coupling THz waves with neural targets and aqueous networks. Section 3 delineates the engineering architecture and communication protocols of the mesoscopic liquid network designed to counter tissue water absorption. Section 4 evaluates the capacity of this network to resolve existing BCI performance deadlocks across typical clinical scenarios. Finally, Section 5 outlines future applications and impending engineering challenges. The literature discussed in this review was primarily retrieved from Web of Science, PubMed, and Google Scholar databases, focusing on peer-reviewed articles published to ensure a comprehensive synthesis of the latest advancements. A graphical overview of the proposed architecture, highlighting the core physical parameters and multidimensional integration strategies, is presented in Fig. 1 and Table 1.

**Fig. 1:**
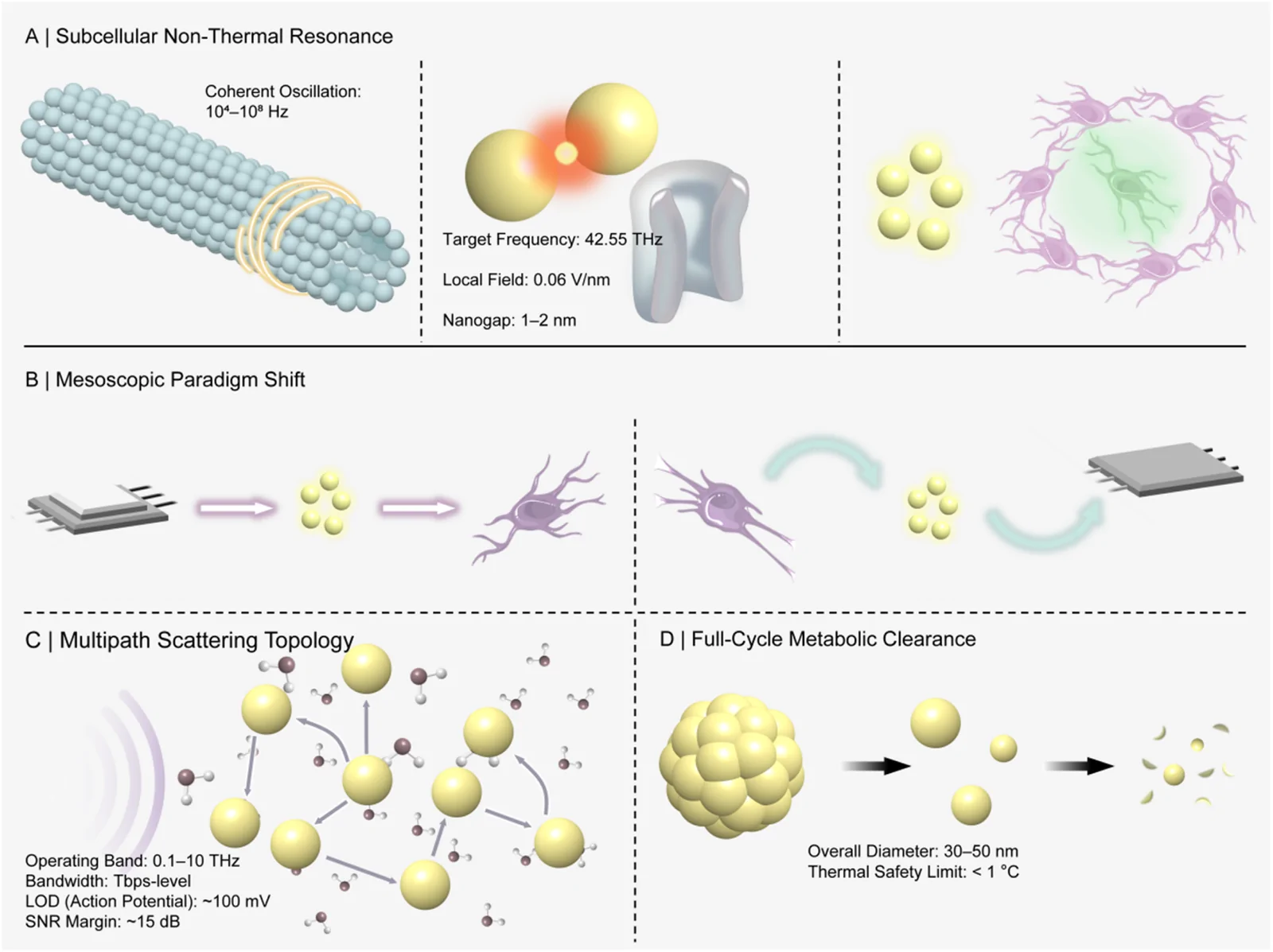
Graphical overview of the terahertz liquid network architecture and core operational parameters. (A) Mechanisms for subcellular non-thermal resonance, highlighting cytoskeletal coupling and targeted ion channel gating devoid of macroscopic thermal damage. (B) The structural paradigm shift from rigid macroscopic implants to free-floating mesoscopic nodes. (C) Non-line-of-sight multipath scattering protocols to bypass aqueous absorption, securing Tbps-level bandwidth and a ∼100 mV LOD. (D) Mesoscopic geometric constraints (30–50 nm) ensuring ultimate metabolic clearance and preservation of the <1 °C thermal safety limit.

**Table 1:** System-level performance comparison highlighting the advantages and limitations of existing brain–computer interface (BCI) architectures *versus* the proposed terahertz liquid network

Architecture type	Spatial resolution/precision	Biocompatibility	Thermal & bandwidth constraints
Non-invasive BCI	Restricted spatiotemporal resolution	Non-invasive	Constrained by the low-pass filtering effect of high-impedance cranial bone
Rigid microelectrode arrays	Elevates signal gain	Mechanical mismatch persistently provokes neuroinflammation and reactive gliosis	Glial scars precipitate irreversible attenuation of chronic recording signals
Flexible polymer electrodes	Scales channel capacities to the thousands	Mitigates mechanical mismatch	Escalating RF power consumption readily breaches the 1 °C thermal safety threshold
THz liquid network (proposed)	Precise, subcellular-level interventions	Free-floating relay nodes fundamentally abolish mechanical shear stresses	Zero-power backscattering fulfills Tbps-level absolute bandwidth without macroscopic heating

## 2. Non-thermal resonance mechanisms coupling terahertz waves with neural targets and aqueous networks

To circumvent the chronic immune rejection^4^ and mechanical constraints^3^ inherent to physical-contact electrodes, the terahertz (THz, 0.1–10 THz) spectrum offers a non-invasive modulatory pathway^9^ that is fundamentally grounded in molecular resonance.^17^ This mechanism relies on the energetic overlap of THz photons (0.4–41.4 meV) with the low-frequency skeletal vibrational modes and hydrogen-bond energy levels of biological macromolecules.^10^ The fundamental energy coupling mechanism bypasses macroscopic thermal heating, functioning instead through microscopic, non-thermal resonance targeting specific chemical bonds within subcellular structures.^10,17^ At the cytoskeletal level, the coherent rearrangement of dipoles within tubulin dimers drives long-range coherent oscillations across the 10^4^–10^8^ Hz spectrum.^10,18^ Normal mode analyses mathematically identify four intrinsic THz-band vibrational modes of microtubules—axial, bending, torsional, and buckling^19^—which subsequently generate nanoscale electromagnetic interactions.^20^ Theoretical models indicate that microtubule mechanical vibrations spontaneously generate nanoscale electromagnetic fields and THz radiation.^20^ This high-frequency electromagnetic coupling potential is empirically supported by scanning tunneling microscopy data, indicating that external electric fields provoke significant dipole flipping in tubulin, manifesting ultrafast, multi-level memory switching characteristics.^21^ These physical responses establish the material basis for non-chemical synaptic information processing.

Regarding membrane potential and channel dynamics, molecular dynamics simulations reveal that targeted 42.55 THz radiation induces symmetric contraction vibrations of carboxyl groups within the calcium channel selectivity filter.^17,22^ Driven by the stark dielectric contrast between the aqueous medium (*ε*_w_ ≈ 80) and the lipid membrane (*ε*_m_ ≈ 2), the localized transmembrane electric field undergoes significant amplification, increasing by a factor of approximately 40.^23^ Mediated by this physical focusing effect, a low-level macroscopic external field (15 kV cm^−1^) translates into a microscopic local field of 0.06 V nm^−1^ at the channel protein,^23^ directly depressing the free energy barrier for transmembrane calcium flux.^22^ Furthermore, when coupled with specific picosecond-pulsed irradiation protocols,^24^ the maximum localized tissue temperature elevation remains strictly below 0.65 K, safely avoiding thermal stress thresholds and precluding macroscopic thermal damage.^23^ Within the biological microenvironment, structured water networks function as essential coupling mediators for THz modulation.^10^ Confined water clusters (*e.g.*, pentagonal dodecahedrons) manifest collective vibrational modes in the 1–6 THz band, coupling directly with protein folding dynamics.^25^ Terahertz coherence spectroscopy identifies long-range synergistic effects within ion hydration layers: highly hydrated multivalent ions in tissue fluid (*e.g.*, high-charge-density Ca^2+^ or Mg^2+^) utilize strong electrostatic coupling to lock up to a dozen peripheral water molecules into a slow-reorientation state, orchestrating an extended, semi-rigid hydrogen-bonded network.^26^ By remodeling the dynamic conformation of these hydration layers, THz radiation indirectly modulates the aggregation of surface signaling proteins and the gating states of ion channels.^22,26^

Collectively, these molecular-level non-thermal resonance phenomena validate the utility of THz waves as physical carriers for non-invasive neuromodulation, simultaneously providing the biophysical rationale for introducing mesoscopic plasmonic networks to actively reconstruct internal communication channels (Fig. 2 and Table 2).

**Fig. 2:**
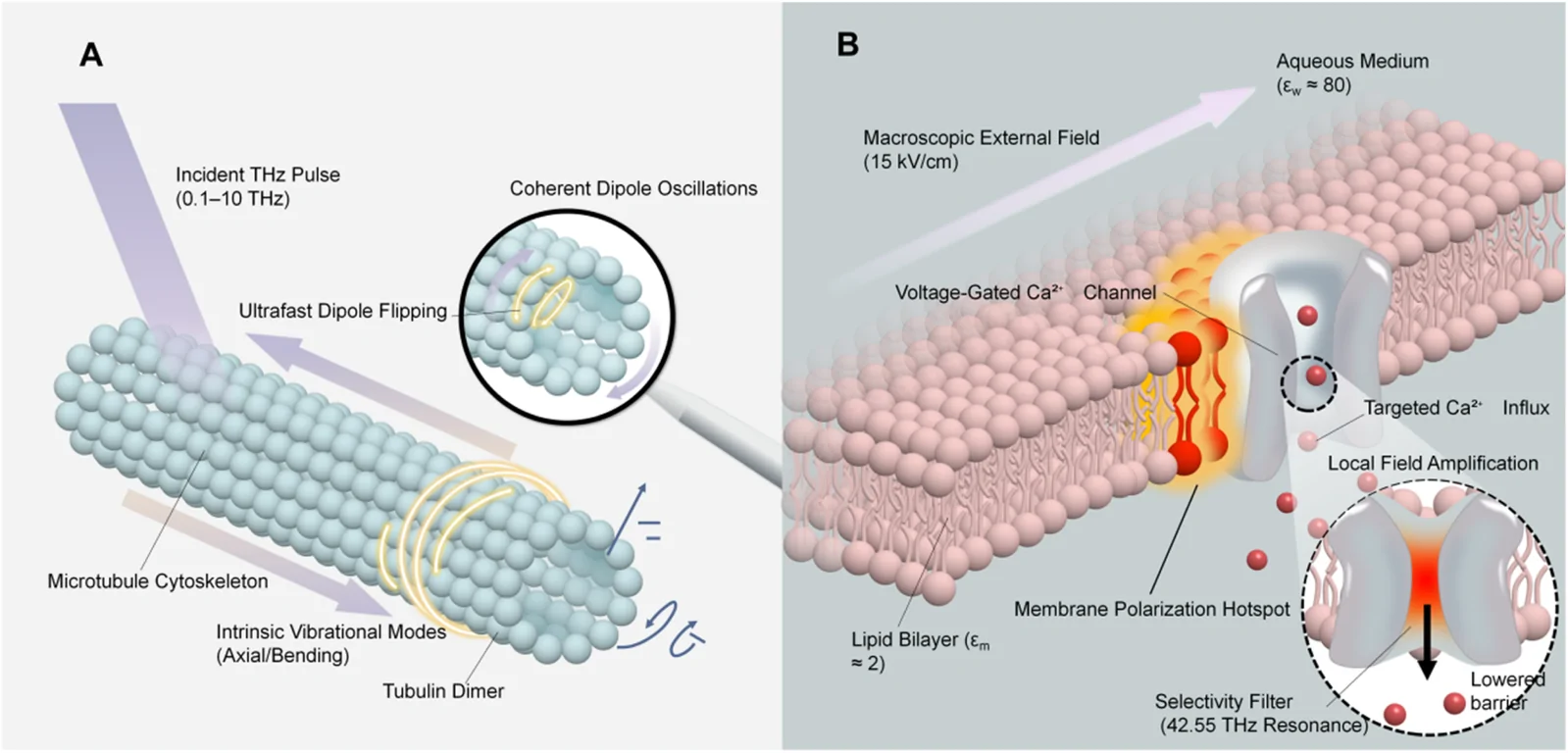
Non-thermal terahertz (THz) resonance mechanisms at the subcellular interface. (A) Cytoskeletal coupling. incident THz pulses induce ultrafast dipole flipping within tubulin dimers, exciting intrinsic vibrational modes (axial, bending, torsional, and buckling) across the microtubule network. (B) Ion channel modulation. The dielectric contrast between the aqueous medium (*ε*_w_ ≈ 80) and the lipid bilayer (*ε*_m_ ≈ 2) generates localized field amplification. Targeted 42.55 THz radiation lowers the transmembrane free energy barrier, facilitating Ca^2+^ influx at a microscopic local field of 0.06 V nm^−1^.

**Table 2:** Summary of THz-induced non-thermal resonance mechanisms across distinct subcellular biological targets

Biological target	Key frequency band	Physical coupling mechanism	Macroscopic physiological effect
Cytoskeletal level (microtubules)	10^4^–10^8^ Hz	Induces ultrafast dipole flipping in tubulin dimers	Establishes the material basis for non-chemical synaptic information processing
Voltage-gated Ca^2+^ channels	42.55 THz	Excites symmetric contraction vibrations of carboxyl groups; amplifies local field to 0.06 V nm^−1^	Depresses the free energy barrier for calcium flux devoid of macroscopic current overflow
Aqueous networks	1–6 THz	Remodels the dynamic conformation of ion hydration layers	Indirectly modulates aggregation of surface signaling proteins and ion channel gating states

## 3. Terahertz liquid network architecture for circumventing aqueous absorption

### 3.1 The aqueous barrier and the communication deadlock of physical-contact implants

The primary physical constraint precluding the integration of THz wireless links *in vivo* is the profound water content of brain tissue, which approaches 70–90%.^27^ Dictated by the intense dipole resonance absorption of THz radiation by dense hydrogen-bonded networks, electromagnetic energy dissipation within the aqueous phase scales exponentially.^10^ Current *in vivo* nanonetwork models (0.1–1.0 THz) establish that classical free-space path loss equations fail entirely against the intense absorption barrier of water-rich tissues.^11^ Constrained by this highly absorptive interstitial fluid, traditional microscale rigid nodes enter a fundamental physical deadlock between transmission range and thermal safety. Forcibly compensating for line-of-sight path loss *via* elevated transmission power precipitates localized energy surges that immediately breach the 1 °C cortical thermal safety threshold, inducing irreversible damage.^8^ Conversely, strict adherence to thermodynamically safe, ultralow-power baselines restricts THz signal propagation to mere hundreds of micrometers prior to complete dissipation, abolishing the physical prerequisites for brain-wide networking.^11^ Independent of these radiofrequency limitations, the chronic implantation of rigid hardware persistently provokes microglia-mediated foreign body responses. The ensuing encapsulation by dense glial scars not only warps the localized dielectric constant distribution but also dictates the progressive histological degradation of the communication link.^4^

### 3.2 Active channel reconstruction: the mesoscopic liquid network paradigm

Surmounting the dual physical boundaries of tissue aqueous absorption and rigid-device immune rejection dictates a structural evolution of the THz BCI from a localized solid-state implant to a mesoscopic liquid network architecture. Adapting principles from nanomedicine and targeted tissue delivery,^28^ this paradigm shifts the strategy for active channel reconstruction away from exploiting narrow THz water-transmission windows. Instead, functionalized metallic capsule microparticles are delivered intravenously or *via* minimally invasive routes to establish *in situ* passive mesoscopic relays within the aqueous brain tissue, entirely replacing conventional micro-tethered flexible electrodes^3^ with biologically functionalized plasmonic architectures.^29^ Under this architecture, biocompatibly modified microcapsules exploit cerebrospinal fluid circulation or microvascular networks to diffuse and selectively anchor within the neural interstitial spaces of the target pathology.^28^ Devoid of active RF transmission circuitry, these highly conductive microparticles function purely as a clustered, passive mesoscopic relay. The infiltration of these massive discrete metallic nodes reconfigures the intrinsically continuous, highly dissipative macroscopic aqueous environment into a discrete mesoscopic electromagnetic network at the subwavelength scale. Histologically, this architecture abolishes the chronic mechanical shear damage associated with rigid devices, while electromagnetically rewriting the fundamental THz wave propagation equations within dense, highly dissipative media *via* the localized modulation of dielectric properties. The evolutionary roadmap of BCI architectures is illustrated in Fig. 3.

**Fig. 3:**
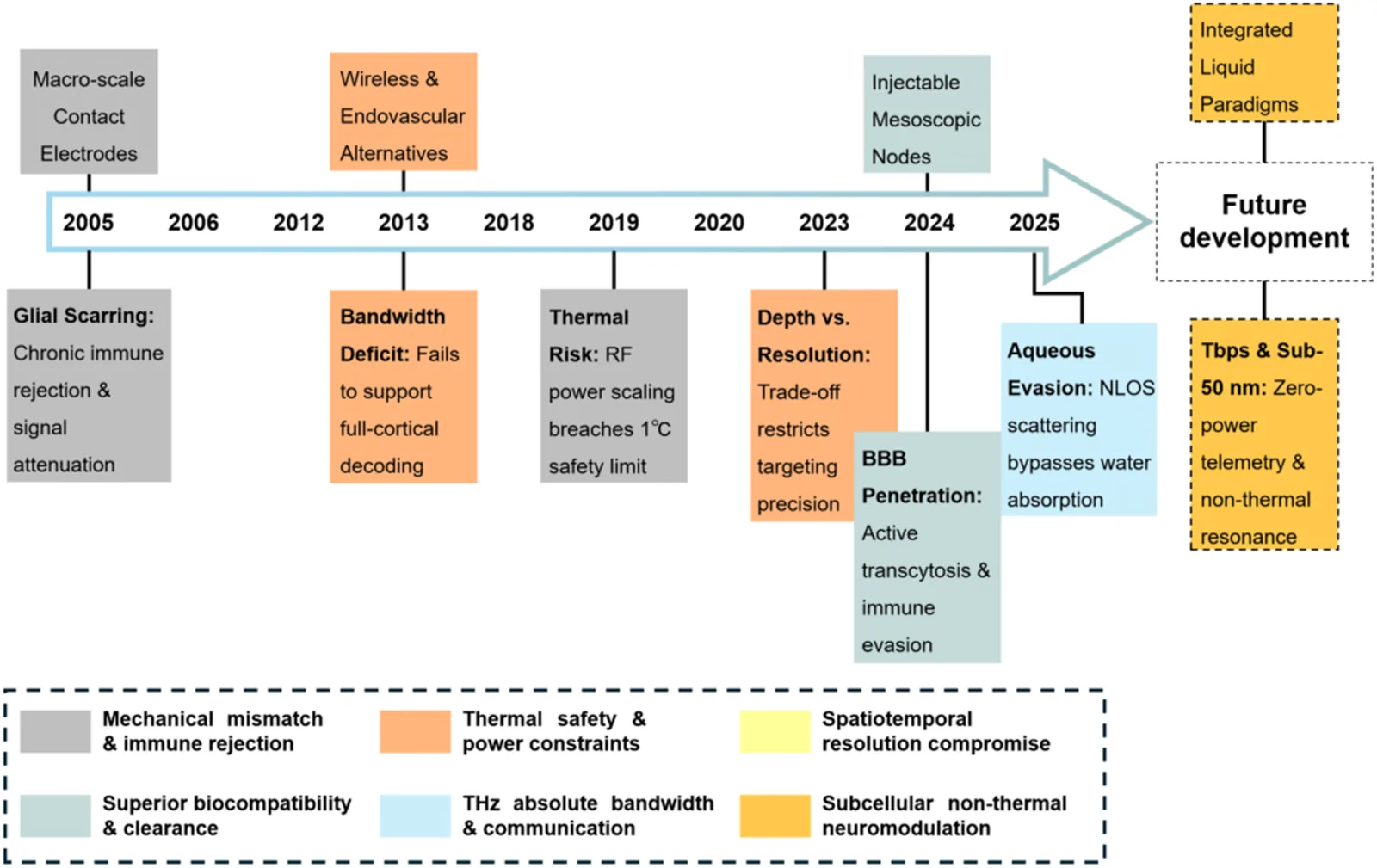
Evolutionary roadmap from macroscopic solid-state implants to the terahertz liquid network paradigm.

### 3.3 Triple aqueous evasion mechanisms and asymmetric communication protocols of the mesoscopic liquid network

The foundational premise of the mesoscopic liquid network lies in circumventing the exponential THz attenuation inherent to water-rich tissues *via* physical channel reconstruction.^10,27^ Exploiting the immense scattering cross-sections of metallic nanocapsules,^30^ this architecture establishes a three-dimensional random scattering topology. This configuration utilizes non-line-of-sight multipath propagation^31^ to physically bypass regions of elevated water content within the neural tissue.^32^ At the microscopic interface, localized surface plasmon resonance (LSPR) on the particles dictates a dual-modal energy transduction. By precisely engineering the geometric parameters of these nanoparticles—such as their aspect ratios and inter-particle nanogap dimensions^30^ —their LSPR absorption bands are selectively tuned^33^ to completely match specific THz excitation windows (*e.g.*, 42.55 THz for calcium channel gating or 0.1–1.0 THz for backscattering). Triggered by this spectral alignment, ultrashort THz pulses undergo *in situ* photothermal conversion^34^ into low-attenuation, high-frequency mechanical waves.^34^ This mechanism effectively traverses the acoustic impedance barrier of the skull^35^ while simultaneously localizing mechanical excitation at the subcellular scale.^34^ Concurrently, the geometric apices and nanogaps of these particles circumvent the electromagnetic diffraction limit,^36^ generating localized field enhancements by two to four orders of magnitude.^37^ This engineered energy compensation ensures the residual electric field at the target interface satisfies the 0.06 V nm^−1^ threshold required for downstream ion channel gating^22,23^ (Fig. 4).

**Fig. 4:**
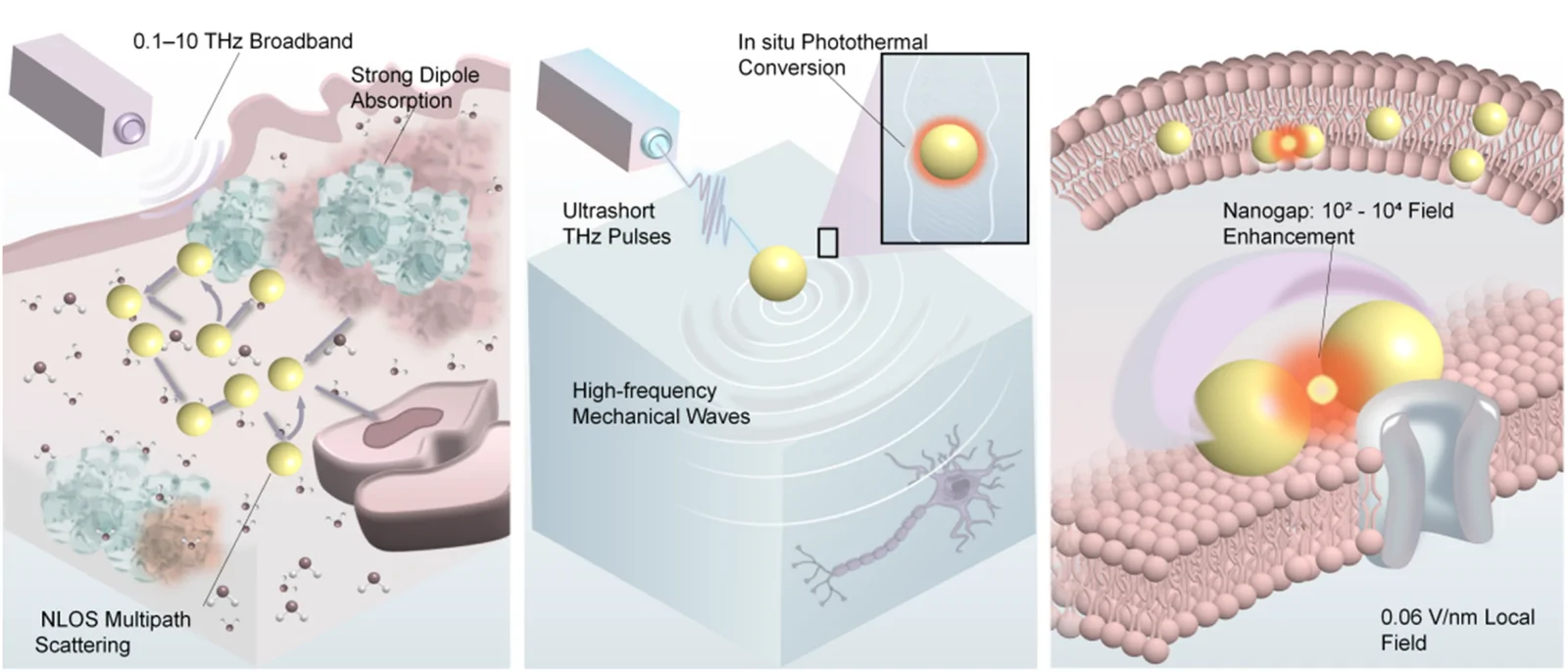
Mesoscopic liquid network strategies for circumventing tissue aqueous absorption. (A) Non-line-of-sight multipath scattering. Dispersed plasmonic capsules (30–50 nm overall diameter) establish a random scattering topology to bypass highly absorptive water clusters. (B) Cross-modal energy transduction. Ultrashort THz pulses trigger localized surface plasmon resonance (LSPR), driving *in situ* photothermal conversion into high-frequency mechanical waves to traverse the acoustic impedance barrier. (C) Near-field enhancement. Strict 1–2 nm nanoscale gaps between the clustered particles amplify the residual electric field by two to four orders of magnitude, reaching the threshold required for targeted ion channel gating.

Regarding communication protocols, the system employs a highly asymmetric, bidirectional cross-modal architecture. The downlink relies solely on extracorporeal THz pulses as active excitation sources, driving intracerebral particles to execute energy transduction and field enhancement, thereby directly precipitating non-thermal resonance in voltage-gated ion channels.^17,22,23^ The uplink abolishes active RF emission entirely, relying instead on a zero-power backscattering mechanism. Membrane potential inversions generated during neuronal firing transiently modulate the Fermi level of a two-dimensional material coating,^38^ thereby perturbing the localized radar cross-section (RCS) of the nanoantennas.^39^ Demodulation of these microscopic RCS perturbations by an external base station sustains high-throughput data backhaul *via* passive backscattering.^40^ Functionally, the limit of detection (LOD) for this cross-modal uplink is defined by the ∼100 mV transient membrane inversions characteristic of single-neuron action potentials. To extract these micro-volt level backscatter shifts from background tissue noise, the physical layer must secure a minimum operational signal-to-noise ratio (SNR) margin of approximately 15 dB.^1,41^ To satisfy this threshold and mitigate uplink SNR degradation dictated by bidirectional path loss, the architecture introduces an asymmetric link compensation mechanism. Intracorporeal nodes employ plasmonic nanoantennas to augment the RCS,^39^ whereas the extracranial base station utilizes massive MIMO phased arrays to secure high-gain reception.^42^ This physical-layer synergy guarantees high-throughput demodulation independent of active internal transmission power.

Securing the reliable extraction of backscattered signals from the highly absorptive and dynamic aqueous brain parenchyma dictates rigorous physical-layer calibration. Within the domain of integrated THz microfluidics, quasi-optical sensors exploit mode-parity-dependent field interactions to execute intrinsic self-calibration, thereby isolating target responses from continuous background fluctuations in capillary-confined analytes.^41^ Translating these quasi-optical calibration principles into the baseband equalization algorithms of the extracranial base station establishes a definitive mechanism to counteract signal-to-noise ratio (SNR) degradation. This architectural adaptation ensures continuous, high-fidelity *in vivo* signal demodulation, rendering the uplink resilient to localized microenvironmental drifts.

Integrating the photoacoustic effect does not compromise the necessity of THz as the primary carrier. Dictated by the Shannon–Hartley theorem, the restricted bandwidth of MHz-spectrum medical ultrasound fails to accommodate the massive data throughput demanded by full-cortical decoding; the absolute bandwidth of the THz regime remains the sole dielectric substrate capable of supporting Tbps-level concurrency.^9^ Unlike conventional focused ultrasound, which is inherently constrained by millimeter-scale acoustic focal spots and cranial aberration,^12,43^ THz radiation translates nanoparticles into *in situ* “nanoscale point acoustic sources”, shrinking spatial interventions to the subcellular level.^34^ Fundamentally, generic macroscopic mechanical waves lack the specificity to couple with designated chemical bonds. Conversely, specific THz photon energies directly resonate with target molecules—exemplified by 42.55 THz targeting calcium channel carboxyl groups—endowing the system with molecular-level modulatory precision.^17,22,44^

### 3.4 Trans-barrier delivery and full-cycle metabolic clearance of mesoscopic nodes

Clinical translation of mesoscopic liquid networks mandates navigating three extreme physiological boundaries: penetrating the blood-brain barrier (BBB), which inherently intercepts over 98% of foreign agents;^45^ resisting glymphatic washout to preserve targeted spatial topology ^46^ and executing complete systemic clearance post-intervention to preclude toxicological accumulation.^47^ To ensure the two-dimensional graphene coating remains intact under the continuous fluidic shear stress of cerebrospinal fluid circulation, the conformal PEG corona acts as a physical shield. It anchors the graphene interface, preventing shear-induced exfoliation and premature degradation before the nodes are metabolically cleared.^28^ Concurrently, conjugated outer-layer targeting peptides, such as the RVG peptide, initiate receptor-mediated transcytosis, actively breaching the BBB.^28^ Post-penetration, the PEG corona impedes non-specific adhesion to the extracellular matrix, facilitating initial parenchymal diffusion.^28^ Triggered by the specific biochemical cues of the target microenvironment (*e.g.*, localized mild acidosis), these discrete particles undergo *in situ* self-assembly into micrometer-scale superstructured clusters.^48^ This conformational phase transition expands their hydrodynamic radius far beyond the 38–64 nm geometric limits of the brain parenchymal interstitium, physically obstructing reverse glymphatic clearance and establishing stable, long-term mesoscopic residency. To preclude heavy-metal toxicity associated with chronic retention, the mesoscopic relays feature a degradable architecture, encapsulating ultra-small primary gold nanoparticles (<5 nm) within a stimulus-responsive polymeric scaffold.^49^ Upon termination of the clinical intervention, specialized external stimuli govern the hydrolysis of this scaffold (50). Dictated by their geometric dimensions falling strictly beneath the ∼5.5 nm physiological limit for glomerular filtration, the *in situ* depolymerized nano-fragments passively follow cerebrospinal fluid efflux and meningeal lymphatic pathways away from the brain,^47^ ultimately entering systemic circulation to achieve complete renal clearance.^50^ This dynamic assembly-disassembly cascade solidifies a histologically compatible, closed-loop metabolic life cycle.

### 3.5 Macro-micro asymmetric system architecture and engineering feasibility

The *in vivo* engineering translation of the mesoscopic liquid network is contingent upon an asymmetric physical architecture that strictly decouples high-power active components from passive intracorporeal nodes. Dictated by the extreme volumetric and energetic constraints imposed on intracerebral nanonodes,^51^ the burdens of large-scale THz carrier generation and highly concurrent computational processing are entirely displaced to an extracranial aggregator base station. This base station exploits CMOS harmonic extraction techniques to push fundamental silicon frequency limits,^52^ utilizing specific N-push oscillator topologies to stably synthesize high-purity THz carriers.^52^ Conversely, intracorporeal nodes harness the surface plasmon polariton characteristics of two-dimensional materials, such as graphene, to dictate extreme antenna miniaturization,^53^ enable dynamic beam scanning,^54^ and secure *in vivo* frequency reconfigurability.^33^ Regarding the emission protocol, the base station implements a multi-band collaborative or broadband picosecond-pulsed strategy: the 0.1–1.0 THz low-frequency window^52^ mediates low-attenuation power transfer and backscatter communication, while femtosecond/picosecond laser-driven photonic THz sources,^52^ concurrently generate ultra-broadband pulses encompassing high-frequency components (*e.g.*, 42.55 THz) to precisely trigger deep-tissue, molecular-level non-thermal resonance. Although complex baseband equalization algorithms inherently introduce computational latency, the integration of ultra-low-latency, edge-side artificial intelligence acceleration hardware ensures that end-to-end intent decoding latency remains constrained within clinically acceptable millisecond thresholds.

To preclude the THz signal shielding inherent to conventional metallic enclosures, the system packaging utilizes highly electromagnetically transparent, biocompatible polymers, specifically adopting liquid crystal polymer (LCP) for subdermal packaging compatibility^55^ and polytetrafluoroethylene (PTFE) for its optimal dielectric parameters.^56^ This low-attenuation encapsulation strategy effectively obstructs biofluid ingress, preserving hardware physical integrity within harsh, hyperthermic physiological environments. Concurrently, governed by the intrinsically low loss tangent of these materials, the localized tissue thermal elevation induced by near-field RF radiation is modeled according to body area network parameters^56^ and rigorously constrained strictly below the absolute 1 °C thermal safety threshold.^8^ Ultimately, this asymmetrically decoupled macro-micro architecture establishes the engineering baseline required for the *in vivo* translation of liquid networks under stringent physiological constraints (Fig. 5).

**Fig. 5:**
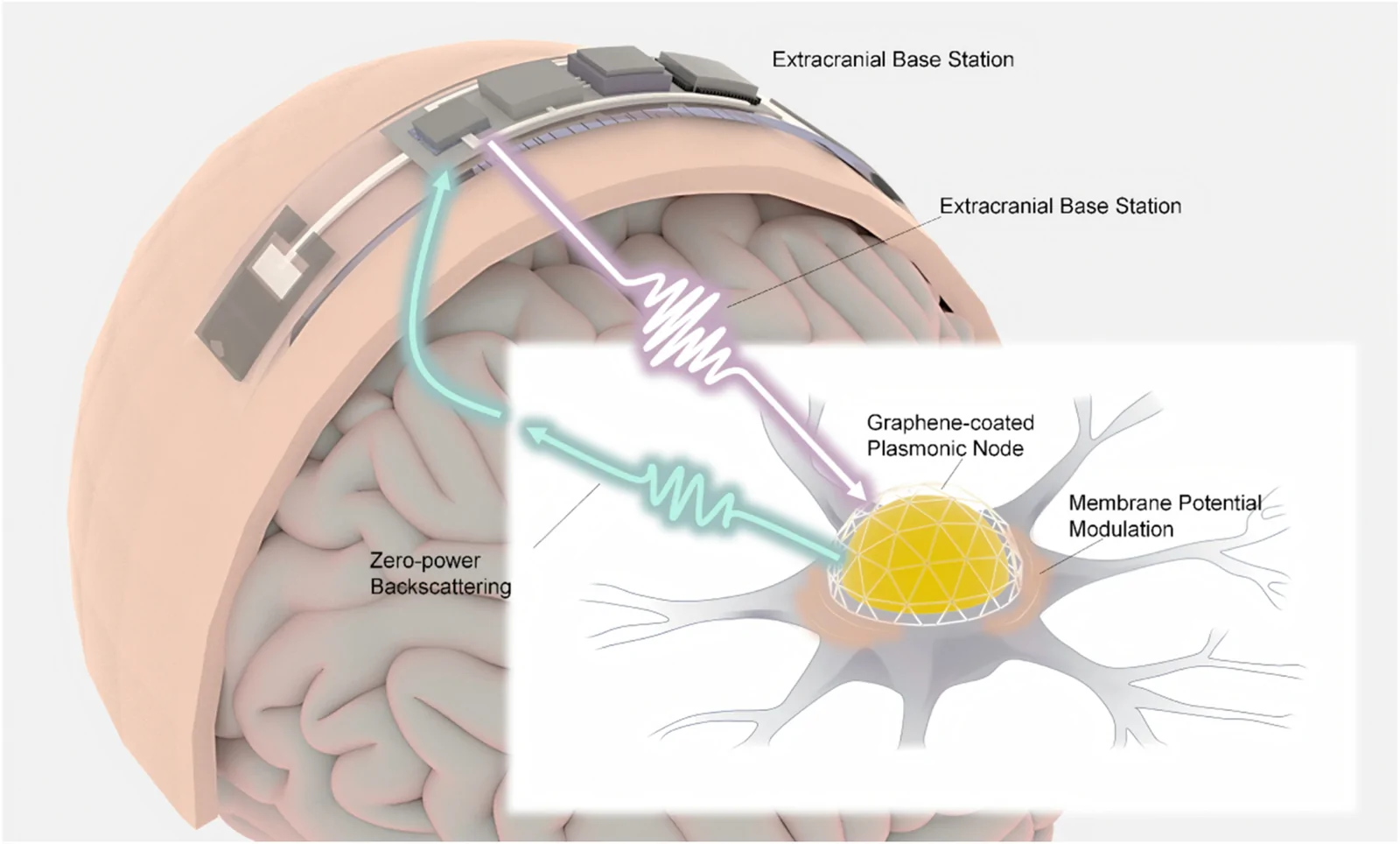
Macro-micro asymmetric architecture and zero-power communication protocol. An extracranial base station transmits active THz pulses for deep-tissue excitation (downlink). Within the brain parenchyma, passive graphene-coated plasmonic nodes (30–50 nm in diameter) interface with target neurons. Neuronal firing modulates the graphene Fermi level *via* transient membrane potential shifts, enabling high-throughput data backhaul through zero-power backscattering (uplink) without internal radiofrequency power consumption.

## 4. Clinical applications

### 4.1 Bottlenecks of existing brain–computer interface architectures in typical clinical scenarios

Constrained by the physical boundaries of bioelectronic interaction, existing brain–computer interface (BCI) technologies encounter fundamental engineering and physiological limitations during clinical translation. Specifically, non-invasive paradigms fail to circumvent the low-pass filtering effect imposed by high cranial impedance;^1^ whereas invasive architectures^2^ remain inherently restricted by foundational material-tissue mismatch constraints^3^ and chronic immune rejection.^4^ These foundational architectural deficiencies manifest prominently within two representative clinical paradigms: audiovisual perception reconstruction and physical neural injury rehabilitation. In the context of audiovisual perception reconstruction for sensory deficits, reproducing high-resolution visual imagery and complex auditory features dictates the highly concurrent processing of tens of thousands of independent information channels.^6^ Constrained by detection depth, existing non-invasive paradigms^1^ and recent intravascular interventional interfaces^2^ fail to achieve single-neuron spatial resolution. Conversely, employing high-density flexible polymer implant arrays within traditional radiofrequency communication architectures precipitates an exponential escalation in transmission power consumption commensurate with channel scaling. Dictated by the restricted thermal convection within the brain parenchyma, this exacerbated heat dissipation readily breaches the 1 °C clinical safety exposure threshold,^8^ thereby thermodynamically capping system bandwidth. Expanding into higher frequency bands to miniaturize hardware dimensions offers no viable alternative; high-frequency electromagnetic waves encounter intense dipole resonance absorption^10^ and exponential signal dissipation within the water-rich neural tissue,^11^ precluding the establishment of high-throughput communication links.

Regarding physical neural injury rehabilitation following stroke or high-level spinal cord trauma, systems depend heavily upon stable, chronic bidirectional electrophysiological reading and writing to compensate for or remodel damaged neural circuits. Sustaining this long-term closed-loop control is obstructed by the mechanical mismatch characteristic of physical-contact electrodes,^3^ which persistently provokes surrounding immune rejection.^4^ The subsequent encapsulation by dense reactive glial scars dictates both a dynamic drift in interfacial bioimpedance and the progressive attenuation of underlying recording signals.^4^ To penetrate this insulating scar tissue and preserve effective stimulation thresholds, conventional systems must passively elevate the writing voltage. This compensatory escalation invariably provokes current overflow, precipitating the aberrant activation of adjacent, non-targeted neural circuits.^57^ Fundamentally, the macroscopic physical fields generated by traditional electrodes lack the spatial specificity required for subcellular intervention, precluding the precise modulation of targeted synapses.^17^ This dual deficit in biocompatibility and micro-modulatory specificity ultimately prevents existing architectures from sustaining high-fidelity, bidirectional closed-loop control over extended clinical durations.

### 4.2 Physical reconstruction of clinical performance bottlenecks *via* the terahertz liquid network

To resolve the multiple physical limitations constraining traditional solid-state BCIs, the terahertz liquid network offers a systematic mechanism for bypassing these bottlenecks at the fundamental biophysical level through active channel reconstruction and macro-micro asymmetric architectural design.

Addressing the intrinsic conflict between bandwidth and thermal safety in audiovisual perception reconstruction, the liquid network employs a passive backscattering protocol,^40^ displacing the entirety of the RF transmission power burden to the extracranial base station. This design precludes the cortical heat dissipation inherently associated with high-concurrency channel arrays, guaranteeing strict compliance with the 1 °C clinical thermal safety threshold.^8^ while sustaining massive communication throughput. Concurrently, to counteract the intense dipole absorption of high-frequency electromagnetic waves by water-rich brain tissue, the dispersed high-conductivity nanoparticle clusters act as subwavelength relays^30^ to induce pronounced non-line-of-sight multipath scattering.^31^ This three-dimensionally distributed mesoscopic scattering topology guides electromagnetic waves around localized regions of high water content, successfully establishing a low-attenuation transmission link deep within the highly absorptive medium.^32^ Exploiting the absolute physical bandwidth of the THz spectrum—which exponentially exceeds that of traditional microwaves—this architecture physically circumvents the constraints imposed by the Shannon–Hartley theorem at lower frequencies, thereby supplying the Tbps-level absolute bandwidth requisite for decoding high-resolution artificial vision and complex auditory features.

Addressing the dual bottlenecks of interfacial impedance and modulatory precision in physical neural injury rehabilitation, the liquid network relies on minimally invasive, injectable microcapsules to fundamentally eradicate the mechanical mismatch between conventional rigid implants and soft neural tissue.^28^ By constraining the geometric footprint of these functionalized nodes to sub-50 nm dimensions, the system physically bypasses the half-wavelength diffraction limit that restricts classical radiofrequency links. This spatial confinement improves the operational resolution of neuro-interventions from the sub-millimeter scales typical of macroscopic arrays down to the single-synapse, subcellular level.^33,44^ Operating as free-floating mesoscopic nodes within the interstitial space, these particles histologically bypass the cascade of chronic immune rejection and dense glial scar formation. This evasion guarantees the long-term stability of interfacial bioimpedance and secures the high-fidelity transduction of bidirectional electrophysiological signals.^4^ Whereas traditional electrodes are routinely forced to escalate stimulation voltages to penetrate insulating scars—precipitating non-targeted current overflow—the liquid nodes resolve this limitation by exploiting plasmonic extreme energy concentration^37^ and near-field THz enhancement mechanisms.^36^ Capitalizing on the unique dielectric alignment between THz photon energies (0.4–41.4 meV) and the low-frequency skeletal vibrational modes of biological macromolecules,^10^ the polarization hot spots generated on the particle surfaces concentrate extremely low-level macroscopic external radiation. This highly localized energy is sufficient to directly induce the molecular resonance of specific chemical bonds at the subcellular scale, effectively amplifying the local microscopic field^23^ to depress the transmembrane free energy barrier of voltage-gated ion channels.^22^ Substituting extensive macroscopic charge injection with targeted, non-thermal resonance significantly mitigates the physiological adverse effects associated with current overflow, establishing a definitive biophysical foundation for precise neural remodeling at the synaptic level.^17^

### 4.3 Summary of clinical applications

Synthesizing the aforementioned analyses of audiovisual perception reconstruction and physical neural injury rehabilitation elucidates the multidimensional superiority of the terahertz liquid network in circumventing the foundational bottlenecks of extant BCIs. Physically, this architecture leverages immense nanoparticle scattering cross-sections^30^ to establish non-line-of-sight multipath propagation^31^ that bypasses aqueous absorption,^32^ ultimately reconfiguring intracorporeal communication channels *via* passive backscattering^40^. This physical channel reconstruction sustains high-speed absolute bandwidth while rigorously constraining system thermal elevation beneath the 1 °C tissue safety threshold.^8^ Histologically, the free-floating mesoscopic morphology mitigates structural shear stress,^28^ fundamentally abolishing the glial scar encapsulation inherent to rigid substrates,^4^ facilitating targeted local field amplification^23^ to alter transmembrane permeability,^22^ achieving non-thermal modulation at the subcellular scale.^17^ Dictated by this cross-scale asymmetric design, the physical substrate of bioelectronic interaction undergoes a substantive transition from a macroscopic active solid-state to a mesoscopic passive liquid-state. This paradigmatic shift securely navigates stringent *in vivo* physiological constraints, establishing a highly translatable engineering trajectory for future systems to achieve ultra-high-throughput, chronically stable, bidirectional closed-loop control (Fig. 6).

**Fig. 6:**
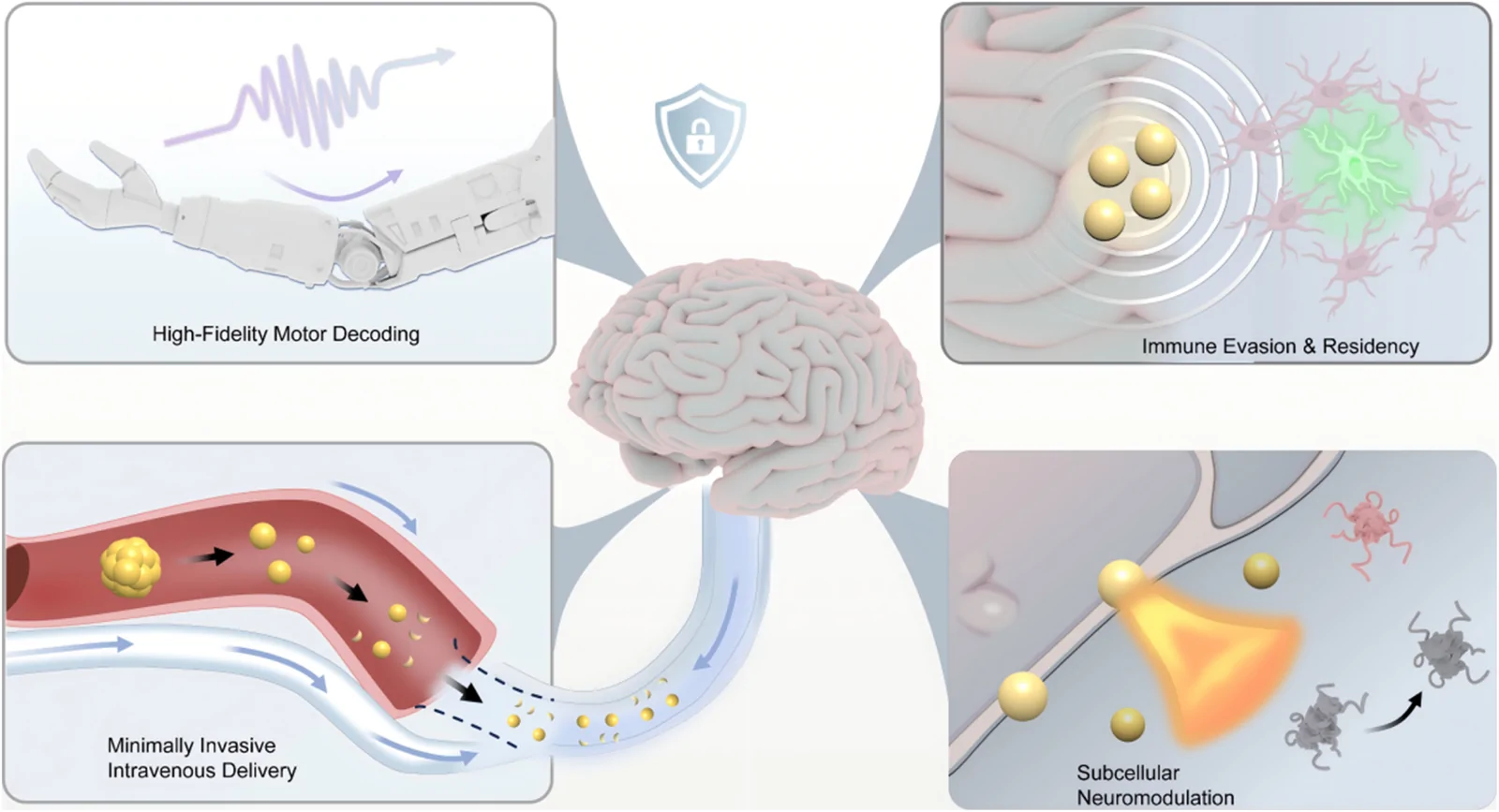
Conceptual overview and clinical translation paradigms of the terahertz liquid network. The architecture seamlessly integrates minimally invasive intravenous delivery, immune evasion, high-fidelity motor decoding (uplink), and subcellular neuromodulation (downlink), resolving the fundamental physical and physiological deadlocks of conventional BCI systems.

## 5. Conclusions and perspectives

### 5.1 Conclusion

Recognizing the inherent physical barrier separating active rigid devices from the soft central nervous system remains prerequisite to initiating a fundamental shift in bioelectronic interaction (4). The terahertz liquid network architecture delineated herein provides a systematic framework to bypass this dual histological and physical deadlock. By employing minimally invasive, free-floating plasmonic nanoparticles as passive relays, this architecture histologically abolishes the mechanical shear stress associated with rigid substrates^3^ and prevents the subsequent chronic immune rejection characteristic of traditional solid implants;^4^ electromagnetically, it exploits THz-photoacoustic cross-modal transduction to penetrate the intense absorption barrier of water-rich neural tissue^34^ and establishes external communication *via* passive backscattering,^40^ entirely externalizing the RF heat dissipation burden associated with extreme data throughput to an extracranial base station (7). This macro-micro asymmetrically decoupled design secures exceptional biocompatibility. Dictated by localized field enhancement, the system utilizes targeted non-thermal resonance to refine the spatial specificity of bidirectional interaction—transitioning from macroscopic charge sensing to subcellular molecular-dynamic intervention (13). Ultimately, this mechanistic advancement severs the mutually restrictive system-level deadlock among bandwidth, thermal safety, and modulatory precision. Consequently, it establishes a definitive physical baseline and engineering blueprint for next-generation clinical BCIs, integrating Tbps-level concurrent communication with molecular-level disease-modifying capabilities.

### 5.2 Perspectives

The primary advantage of the terahertz liquid network lies in its capacity to fundamentally resolve the mutually antagonistic bottlenecks of conventional BCIs: it abolishes mechanical shear stress, circumvents the 1 °C thermal safety limit *via* passive backscattering, and elevates targeted spatial resolution to the subcellular scale. However, assessing the ultimate clinical impact of this architecture requires acknowledging several inherent limitations. Transitioning to true *in vivo* clinical applications dictates that the system must navigate stringent physical and physiological boundaries. Specifically, the primary limitation stems from the chronic histocompatibility of the mesoscopic nodes. Although the intrinsic glymphatic pathway^46^ provides a theoretical closed-loop for the physical excretion of nanostructures,^47^ genuine clinical translation depends upon degradable vectors possessing both targeting affinity and microenvironmental responsiveness.^48,49^ This necessity dictates the precise quantification of pharmacokinetic models governing the initial evasion of biological barriers,^28^ receptor-mediated transcytosis across the BBB,^28^ and the ultimate reverse transport of nano-fragments away from this restrictive interface,^45^ thereby fundamentally eradicating the chronic toxicological risks posed by heavy-metal accumulation within endogenous neural circuits. Securing high-fidelity decoding across the intact cranium remains a major challenge. While intracerebral liquid relays bypass the strong absorption of parenchymal water,^34,40^ their reliance on zero-power backscattering introduces new physical vulnerabilities. Specifically, dynamic physiological flows—such as cerebrospinal fluid (CSF) pulsation—drive continuous topological drift of the free-floating nanoparticles. This real-time spatial fluctuation severely degrades the signal-to-noise ratio (SNR), risking the loss of attenuated transcranial return signals within background tissue noise.^27,31,32^ To compensate, the system architecture must be highly asymmetric. Extracranial base stations require the integration of high-purity THz solid-state sources^52^ with tunable phased arrays for dynamic beam focusing.^33,54^ For data processing, robust baseband equalization algorithms are essential to continuously track and calibrate RF phase detuning,^42^ ensuring signal integrity before motor intent is extracted *via* lightweight neural networks. Moving beyond hardware constraints, the ultimate clinical translation of this architecture dictates a paradigm shift in biophysical modeling. Governed by the extreme heterogeneity of the neural microenvironment, classical integer-order equations inevitably fail to capture anomalous electromagnetic diffusion and localized memory effects. Consequently, adopting fractional modeling frameworks^58^ becomes imperative to precisely characterize the nonlinear electro-capacitive dynamics and dielectric boundary interactions of the fluidic nodes. Ultimately, only the synergistic integration of physical-layer noise suppression, fractional electrodynamic calibration, and upper-layer neural decoding can authentically sustain Tbps-level concurrent data backhaul under the absolute constraint of zero intracorporeal power consumption. The ultimate boundary resides in the ethical sovereignty governing neuro-interventions. As modulatory precision advances down to the scale of synaptic clefts and molecular conformations,^17^ this interface undergoes a substantive elevation into an “electroceutical” capable of rectifying underlying biophysical pathologies.^44^ Such profound bidirectional read-write access to fundamental biological processes compels the hardware physical layer to solidify anti-hijacking protocols against advanced neuro-interventions,^57^ ensuring intrinsic neuro-security.^59^ Concurrently, the medical and neuroengineering communities must collaboratively delineate proactive ethical boundaries:^1,57^ An impenetrable jurisprudential demarcation must be established between “compensating for neurological deficits” and “manipulating cognitive liberty”. Only through such rigorous frameworks can this transformative technology safely and rationally transition into the clinical reality of multimodal neuro-therapeutics.

## Author contributions

Chenyan Zhou: conceptualization, methodology, visualization, writing – original draft, writing – review & editing. Xinran Ding: investigation, resources, visualization. Jiamin Zhao: investigation, data curation, formal analysis. Xuanhao Gu: investigation, validation, writing – review & editing. Zhengyang Xu: software, formal analysis, visualization.

## Conflicts of interest

There are no conflicts to declare.

## Abbreviations

BBB: Blood–brain barrier
BCI: Brain–computer interface
CMOS: Complementary metal-oxide-semiconductor
LCP: Liquid crystal polymer
LSPR: Localized surface plasmon resonance
MIMO: Multiple-input multiple-output
PEG: Polyethylene glycol
PTFE: Polytetrafluoroethylene
RCS: Radar cross-section
RF: Radiofrequency
RVG: Rabies virus glycoprotein
SNR: Signal-to-noise ratio
Tbps: Terabits-per-second
THz: Terahertz

## Data Availability

This manuscript is a review article. All information and data discussed in this work were derived from previously published literature, which has been properly cited in the References section. No new primary experimental data were created or analyzed in this study. Therefore, data sharing is not applicable to this article.
